# The influence of olfactory disgust on (Genital) sexual arousal in men

**DOI:** 10.1371/journal.pone.0213059

**Published:** 2019-02-28

**Authors:** Charmaine Borg, Tamara A. Oosterwijk, Dominika Lisy, Sanne Boesveldt, Peter J. de Jong

**Affiliations:** 1 Department of Clinical Psychology and Experimental Psychopathology, Faculty of Behavioral and Social Sciences, University of Groningen, Groningen, the Netherlands; 2 Division of Human Nutrition, Wageningen University, Wageningen, the Netherlands; University of Rome, ITALY

## Abstract

**Background:**

The generation or persistence of sexual arousal may be compromised when inhibitory processes such as negative emotions, outweigh sexual excitation. Disgust particularly, has been proposed as one of the emotions that may counteract sexual arousal. In support of this view, previous research has shown that disgust priming can reduce subsequent sexual arousal. As a crucial next step, this experimental study tested whether disgust (by means of odor) can also diminish sexual arousal in individuals who are already in a state of heightened sexual excitation.

**Methodology:**

In this study, participants were all men (N = 78). To elicit sexual arousal, participants watched a pornographic video. Following 4.30 minutes from the start of the video clip, they were exposed to either a highly aversive/disgusting odor (n = 42), or an odorless diluent/solvent (n = 36), that was delivered via an olfactometer, while the pornographic video continued. In both conditions the presentation of the odor lasted 1 second and was repeated 11 times with intervals of 26 seconds. Sexual arousal was indexed by both self-reports and penile circumference.

**Principal findings:**

The disgusting odor (released when the participants were already sexually aroused) resulted in a significant decrease of both subjective and genital sexual arousal compared to the control (odorless) condition.

**Significance:**

The finding that the inhibitory effect of disgust was not only expressed in self-report but also expressed on the penile response further strengthens the idea that disgust might hamper behavioral actions motivated by sexual arousal (e.g., poor judgment, coercive sexual behavior). Thus, the current findings indicate that exposure to an aversive odor is sufficiently potent to reduce *already present* (subjective and) genital sexual arousal. This finding may also have practical relevance for disgust to be used as a tool for self-defence (e.g., Invi Bracelet).

## Introduction

### Sexual arousal

Sexual arousal has been used to refer to sexual excitation, sexual drive, and sexual motivation [1–3]. Following the dual control model of human sexuality [4], the level of sexual arousal is determined by the interaction of sexual excitatory and sexual inhibitory processes. From such perspective, the generation or persistence of sexual arousal may be compromised when inhibitory processes outweigh sexual excitation. Thus, relatively strong inhibitory tendencies may undermine or interrupt sexual arousal. Many different interpersonal and intrapersonal factors may contribute to inhibitory processes, including a problematic relationship, anxiety and fear. The current study focuses on the inhibitory impact of disgust on sexual arousal.

### Disgust and sexual arousal

Disgust has been conceptualised as a disease avoidance mechanism; via eliciting the overwhelming urge to withdraw from disgusting cues, disgust helps prevent exposure to pathogens [5, 6]. Because intimate/sexual behaviors imply massive pathogen exposure, sexual stimuli and behaviors seem obvious candidates for eliciting disgust [5, 7]. In line with this, it has been shown that sexual by-products (e.g., saliva, sperm) can be strong elicitors of disgust when placed outside of a sexual context [8–10]. Thus from this perspective, it seems that sexual stimuli and behaviors are inherently ambiguous and can elicit both sexual appetite and disgust. To the extent that disgust is the dominant response, this will interfere with the development or persistence of sexual arousal [11]. Specifically, disgust serves an avoidance motivation that interferes with responses (sexual arousal) that serve sexual approach [11]. Consistent with the view that heightened disgust may compromise “healthy” sexual responding; women with an inability of having sexual intercourse have reported higher levels of disgust for stimuli contaminated with sexual by-products than women without sexual problems [12]. Similarly, men with erectile disorder demonstrated lower willingness to approach stimuli contaminated with sexual by-products than men without sexual problems [12].

In contrast to the problems associated with heightened disgust, lowered disgust may be problematic because this may disinhibit the generation of sexual arousal, thereby lowering the threshold for engaging in riskier sex and/or for engaging in coercive sexual behaviors. Pointing to the relevance of lowering sexual arousal in certain situations, there is evidence that heightened sexual arousal can interfere with our decision-making [13–15]. For instance, sexually aroused men have been found to endorse a significantly wider range of sexual activities and statements, when compared to men who were not aroused [13]. In addition, and perhaps even more important, this also applied to statements concerning sexual coercion such as *‘Would you keep trying to have sex after your date says “no”‘*. In much the same way, sexual arousal was related to sexual violence. Using a hypothetical date-rape scenario, it was found that sexually aroused men considered it more likely to behave in a sexually forceful manner than men who were not aroused [16]. Recent studies using a similar experimental setup showed comparable results [17,18]. Considering these consequences of sexual arousal, identifying strategies to inhibit sexual arousal might be helpful in preventing sexually coercive behaviors or attempts.

### Effect of disgust on sexual arousal

Supporting the view that disgust may inhibit sexual arousal, it has been shown that watching an erotic film resulted in less sexual arousal—as indexed by physiological measurements—when participants (all women) were exposed to disgusting pictures (e.g., pictures of infected bodies, rotten animal parts) prior to the erotic film [19]. In addition, it was found that for both men and women, disgusting priming images reduced the subjective sexual arousal in response to subsequently presented sexually explicit images [20]. This also corresponds to evidence illustrating that olfactory information is an important cue for selecting a sexual mate [21].

Both previous studies provided evidence to indicate that prior exposure to disgusting stimuli attenuates women’s as well as men’s sexual responsivity. An important question that remained unanswered is whether disgust may also affect sexual responsivity when participants are already in a state of sexual arousal. In other words, whether heightened disgust not only interferes with the development but also with the persistence of sexual arousal.

If disgust elicitors can indeed reduce heightened sexual arousal, this would not only be of theoretical relevance but might also have practical implications. Specifically in the context of transgressive sexual behavior it is beneficial if sexual arousal can be inhibited in people, even if they already are in a state of sexual arousal. This more likely mimics real life scenarios involving sexual offenses. As such, it would point to the usefulness of employing disgust elicitors to reduce perpetrators’ sexual excitation within the context of arousal-driven coercive sexual behaviors.

### The current study

Therefore, the major aim of this study was to test the inhibitory effects of disgust on heightened sexual arousal. Because most sexual perpetrators are men [22], it is especially relevant to know whether disgust can reduce sexual arousal in a male sample. Hence, we decided to focus on male participants. In the current study, we not only examined subjective sexual arousal, but also included an objective measure (penile circumference) of physiological arousal. This allowed us to test the impact of disgust on both more explicit and more implicit (reflexive) indices of sexual arousal.

Unlike both of the previous studies on the impact of disgust on sexual arousal, in this study we induced disgust via an offensive odor. In comparison to commonly used visual stimuli [20], odor is generally considered as a stronger and more direct way of eliciting disgust, especially since odors are important signals of harmful stimuli that pose a threat of contamination [23, 24]. Thus in short, this experimental study was designed to examine whether a disgusting odor can diminish men’s subjective and physiological sexual arousal when being in a state of sexual excitation.

## Method

### Participants

Participants were male students (N = 78) (Mean age = 21.1 years, *SD* = 2.1) from the University of Groningen. Before collecting our sample we had decided on a sample size of n = 80 as this would be both an attainable sample size and sufficient in terms of power. As indicated by a power analysis on the basis of Gpower detecting a difference in means (in arousal reduction between active and control condition) by using a t-test would amount to a power of .82 to .94 for a medium to large effect size (d = .50; d = .80) [25]. However, due to time constrains and limitations in the availability of materials, the number of participants was slightly less than the 80 participants we originally aimed for. In addition, not all cases could be included in all analyses. When performed the obtained power in the current sample, it lies between .77 and .94 for medium to large effect sizes (d = .50; d = .80).

The participants were recruited using leaflets and the university credit system, and were allotted a reimbursement of either 20 euros (*n* = 2) or course credits for their participation. Eligibility required, having a sexual preference for women, a clear nose (i.e., without a cold), a normal sense of olfaction, no (self-reported) olfactory problems and no treatment for respiratory diseases (e.g., asthma). These questions were answered on a self-report measure as part of the participation eligibility checklist.

During recruitment, it was conveyed that the purpose of the study was to “*test the effect of different odors on physiological responses*” and that the experiment included watching pornography and penile measurement. In order to prevent expectancy effects, the disgusting nature of the odor was not mentioned. The mentioning of pornography and penile measurements might have resulted in a selection bias, causing more (sexually) inhibited individuals to not participate in this study. Participants were randomly assigned to the experimental (*n* = 42) or the control condition (*n* = 36). The duration of the experiment was approximately one hour. The Ethical Committee of Psychology for University of Groningen approved this study (ECP-code 15086-NE), and all procedures were conducted accordingly.

### Stimuli

#### Baseline video

To ease participants into the experimental setting and create similar baseline rates, participants were shown a pleasant (baseline) comedy video at the start of the experiment. The two and a half minute video consisted of several fragments of the BBC one comedy series ‘Walk on the wild side’. The comedy video and the content of the pornographic video were very distinct (i.e., talking animals, vs. people engaging in myriad of sexual acts). This baseline video could have increased the general arousal with a positive impact on inducing sexual arousal. The main aim of this video clip was to put the participants at ease, as the measurements included in this study could be felt as intrusive (thereby possibly decreasing the effectiveness of the pornographic content).

#### Pornographic video

Sexual arousal/excitation was induced using a ten-minute pornographic video. The video was compiled out of several fragments from recent pornographic material aimed at heterosexual men. The porn-fragments all contained heterosexual interactions between different Caucasian couples (which is the far majority in this part of the country, and in the study sample) and were selected for being free from any out-of-the-ordinary or distracting elements such as music. Scenes in the video featured penile penetration, fellatio, cunnilingus and anilingus. The research team members agreed on the qualities of this video material to elicit sexual arousal. While watching the movie, participants were instructed not to masturbate.

#### Odor

The aversive odor used in the study was a mixture containing multiple disgust elicitors. One of the elements used had some similarities to skunk odor, while other parts were associated with rotten garlic and the odor of smelly feet. The elements combined created a new type of odor that is considered highly disgusting, easily perceivable and yet low in toxicity. The odor was composed and provided by the Dutch company Invi, and toxicologically approved by the ‘Dutch Organization for Applied Scientific Research’ (Nederlandse Organisatie voor toegepast-natuurwetenschappelijk onderzoek, TNO).

Since the compound was deemed to be (too) aversive in its undiluted form, the liquid concentrate was diluted (1:100,000) with propylene glycol, before usage in the olfactometer. Propylene glycol was also used in the odorless control condition. Propylene glycol was chosen because it is almost odorless and safe in use [26].

### Measures

A schematic overview of the structure in which the following measures were used can be found in the supplementary material (S1 Fig).

#### The Disgust propensity and sensitivity scale—Revised (DPSS-R)

This 12-item DPSS-R [27] has shown to be a parsimonious and psychometrically sound measure with predictive validity for actual avoidance behavior [28]. It measures people’s disgust propensity and sensitivity, which described the tendency to react with disgust, and find the experience of this emotion very worrisome, respectively [29]. Both factors consist of six statements such as ‘*I avoid disgusting things*’ (disgust propensity) and ‘*It scares me when I feel nauseous’* (disgust sensitivity) and are rated according to how frequently they apply. Rating occurs on a 5-point Likert scale ranging from never (1) to always (5). In general, both factors are high in internal consistency (Propensity α = .78; Sensitivity α = .77). However, for our sample the internal consistency for disgust propensity (α = .65) and for disgust sensitivity (α = .64) was below than the conventional border of 0.7 and somewhat lower than in previous studies [29]. Yet, as the disgust propensity and sensitive scale is only used to check for differences between groups that might blur the findings, we preferred to keep the scale in the study.

#### Subjective sexual arousal

Sexual arousal was assessed on a 0–100 visual analogue scale (VAS, 0 being “not at all aroused” and 100 being “very much aroused”) after the porn-video; using the following questions; “*What was your peak level of sexual arousal during the movie*?*”*, *“What was your level of sexual arousal directly before the onset of the odor induction”* and *“What was your level of sexual arousal directly after the odor induction*?*”*. These measures were provided only once after the porn video in order not to interfere with the actual arousal manipulation.

#### Subjective emotions

Subjective emotions were measured before and after the porn-video. Participants indicated on a visual analogue scale (VAS) from, 0 “not at all” to 100 “very much” how happy, angry, disgusted, and anxious they felt.

#### Facial disgust response

Facial Electromyography (EMG) was used to measure the muscle activity of the m. *levator labii*, which is a unique physiological indicator of the disgust response (wrinkling of the nose and lifting of the upper lip) [30,31]. Negative affectivity (*m*. *corrugator supercilii*, involved in frowning; [30,31]) was also measured using EMG. However, due to noise in the data from the corrugator muscle, the corrugator responses were not included. This was not considered a problem since the levator is specific to disgust, and the corrugator was added merely as a control measure.

#### Genital sexual arousal

Genital sexual arousal was measured via penile circumference measurement (PCR), specifically Indium-gallium strain gauges were used. These types of gauges are generally considered accurate and valid measures of genital sexual arousal in men, and have been used in previous work [32–35].

### Apparatus and materials

#### Display and task presentation

The study was implemented in E-prime (version 2.0.10.353). A software that was programmed to provide the participants with instructions on how to proceed throughout the study, prompt the presentation of the videos and also provide the visual analogue scales on which participants could register their emotions. In addition E-prime triggered the olfactometer and instigated the recording of the physiological measures (specifically the EMG and the PCR).

The task was presented on a 27-inch monitor and participants were seated at a distance of approximately 85 cm away from this monitor. The video (10 by 20 cm) was set against a black background, and was viewed at a visual angle of 6.73°.

#### EMG

The EMG-electrodes were, placed in pairs on the left side of the participant’s face [36]. NuPrep gel (Weaver and Co., Aurora, CO) was used to scrub the participant’s skin on the sites of measurement and TECA electrode conductivity gel (Oxford Instruments Medical Inc.) was placed inside the caps of the electrodes to ensure a proper conduction of signals. The electrodes measured 6 mm and had a contact surface of 2.8 mm. Double-sided adhesive tape was used to secure the electrodes. EMG-data were recorded using TMSi Polybench (version 2.0).

#### Penile circumference measurement

Indium-gallium strain gauges (Behavioral Technology, Inc) in three sizes were used to measure penile circumference. Changes in tumescence of the penis cause the gauges to stretch, resulting in a changed ability of the indium-gallium core to conduct electricity. Consequently, changes in voltage are indicative of changes in penile circumference. The departmental technical research team built a steel cylinder with several circumference widths to calibrate the gauges. By calibrating several gauges with this steel cylinder, it was calculated that a change of 10,000μV corresponded to a change of 1.6 mm in circumference. Voltages were recorded using TMSi Polybench (version 2.0).

#### Olfactometer

Both odors were presented using an olfactometer (manufactured according to [37]) through which a controlled and temporally precise distribution of odors is possible. An air-compressor provided the olfactometer with a constant airflow, while the olfactometer allowed for the distribution of the aversive and the odorless diluent used as control. The compressor was placed in another room adjacent to where the experiment was conducted separated by a wall of concrete. No objective measurements were taken about whether the noise of the compressor could have been experienced as distracting.

The olfactometer first filters the air from the air compressor to prevent contamination with other odors, and thereafter dispenses the air over multiple channels. The air from these channels consecutively flows through reservoirs containing either the 10 ml of the diluted odor (experimental-condition), 10 ml of propylene glycol (control-condition), or clean air and then flowed on towards the nosepiece inside the participant’s nose.

During the first part of the experiment the olfactometer generated a constant airflow of 0.5 L/min, without the addition of any odorants. After 4.00 minutes of watching the pornographic video, the airflow was increased with 3 L/min. of clean air. Later, at 4.30 minutes, channels used in the olfactometer switched, temporarily replacing the extra 3 L/min of clean air with, depending on the condition, either the disgusting or the odorless diluent. The airflow was increased with clean air prior to induction of the airflow containing the aversive or control odor, to avoid contamination of the onset of the odor with the onset of increased airflow (e.g. mechanical or tactile impact on the nasal mucosa). In both conditions the presentation of the disgusting or odorless diluent lasted 1 second and was repeated 11 times with intervals of 26 seconds (in which clean air was provided). Throughout this period the airflow remained constant at 3.5 L/min.

### Procedure

As soon as they arrived at the lab, instructions about the procedure were provided to the participant. The participant was invited to read and sign the informed consent and to take a place in a comfortable chair in a private part of the lab. A Velcro-belt, used for carrying the nosepieces of the olfactometer, was strapped around the participant’s chest. The EMG procedure was explained, and the electrodes were attached. Subsequently the nosepieces were attached to the Velcro-belt and the experimenter explained how to fit the nosepieces of the olfactometer into the participant’s nose.

Next, the air compressor was turned on in order to let the participant experience the airflow, and the functioning of the EMG-electrodes was checked. The participant was told to go with his emotions and to try to enjoy the movie that was going to be shown. The experimenter put on the headphones for the participant and left him in private with dimmed light. Only then could the participant start the computer-task, which besides showing the participant instructions and the video-material later on, also served to direct the olfactometer and control the recording of physiological measures.

The first instruction brought about by the computer-task informed the participant on how to apply the PCR and how to choose the appropriate size of the gauge. After applying the PCR, the EMG and PCR signals were checked (from the experimenters’ room). The experiment could only continue when both the signal from the EMG and PCR indicated that everything was functioning in order. Then the baseline comedy video was shown to the participant. Before and after this video the participant could indicate his emotional subjective state using VASs. Afterwards the participant could start the porn-video to induce sexual arousal. After 4.30 minutes of pornographic video, the olfactometer started releasing puffs of odor. When the video was finished, the participant used the VAS again to indicate his emotional state. He could then detach the PCR, and when ready he could call the experimenter to request for the detachment of the other devices (e.g., the EMG). Finally, the participant was asked to answer some further questions about how he experienced the odor and his levels of sexual arousal and ended the experiment by filling in the questionnaire package.

### Data handling

All data were processed in SPSS (version 22.2). Most variables used were constructed as difference scores. They were calculated by subtracting a baseline-score, in such a manner that the positive scores indicate an increase, whereas negative scores indicate a decline in the specified variables.

#### Subjective data

For the DPSS-R the items for disgust propensity were constructed by summing the 6 items from this scale (i.e., 1, 4,5,6,8, and 10), the same was done for disgust sensitivity (i.e. 2, 3, 7,9,11, and 12).

For the measurement of subjective-disgust, difference-scores were calculated subtracting the VAS-score obtained before, from the VAS-score obtained after the pornographic video. For the VAS, which measured self-reported sexual arousal, the difference scores of the sexual arousal as experienced before the onset of the odor, were subtracted from the sexual arousal after the onset of the odor. Lastly, it was checked whether the peak value of subjective sexual arousal before the odor, was generally larger than zero, in other words whether sexual arousal was successfully induced.

#### Physiological data

EMG-data were filtered (high-pass 10 Hz; low pass 500 Hz) and visually inspected with the computer programme Aphys (version 2.11.1.0, as constructed by Ruiter, 2017). For analysis, a mean root square was calculated for a baseline period before each puff (-3 to 0 seconds) and a period after every puff (1 to 3 seconds; based on visual inspection). By subtracting the baseline from the latter, difference-scores as a measure for physiological disgust were obtained for further analysis. To avoid results that were contaminated by the shadowing effects of prior puffs, for each participant, only the difference-score of the first puff was taken into account.

PCR data were filtered (1 Hz low-pass filter) and inspected in Aphys, discarding failed trials or parts of trials with artefacts. Data entries that were discarded due to mechanical problems were missing completely at random (See Results section, and the codebook and the data on ‘*link dataverse*’), and thus ignored in the rest of the analysis. Voltage-scores were transformed into millimetres to make them more easily interpretable and comparable to other studies [38]. Two difference score variables were calculated based on the PCR-data. To check whether the pornographic video induced physiological sexual arousal, the physiological sexual arousal during the first part of the pornographic movie was subtracted from baseline physiological sexual arousal during the neutral movie.

Next, we assessed the impact of odor on the physiological sexual arousal. This was done by subtracting the physiological sexual arousal during the first part of the pornographic movie, from the mean sexual arousal during the second part of the pornographic movie (when puffs were being released). Thus the more negative the value, the stronger the disgust-induced inhibition. Subsequently, these difference-scores were used to calculate whether the aversive odor differentially impacted sexual arousal compared to the odorless diluent.

### Data analysis

#### Manipulation checks

We first verified whether there were undesirable pre-existing group-differences in trait disgust that might have influenced the results, by subjecting participants’ sensitivity and propensity scores on the DPSS-R to univariate analyses of variance (ANOVA’s). Thereafter, we carried out a series of manipulation checks. First, any participants that showed a decrease in physiological sexual arousal in response to the pornographic video were removed from analysis, as they would not provide any information about the ability of disgust to reduce sexual arousal. To check the amount of increase in sexual arousal in the rest of the sample (before the experimental disgust manipulation) we tested whether the physiological sexual arousal induction scores (i.e. difference score from the level of physiological sexual arousal during the first part of the pornographic video minus the physiological sexual arousal during the comedy video) differed significantly from zero. And, likewise, whether the peak level of subjective sexual arousal differed significantly from zero. Then, to check whether this increase was similar for the experimental and control group, we subjected the subjective peak level of sexual arousal score and the physiological sexual arousal induction score to an ANOVA. To check whether the experimental disgust manipulation was successful in heightening disgust, subjective and physiological disgust change scores were also subjected to ANOVA’s.

#### Hypotheses testing

To test the hypothesis that the induction of disgust would lower sexual arousal, we subjected the pre-post disgust induction arousal change scores of both conditions to ANOVA’s. However, while ANOVA’s are to a great extend robust against violations of normality [39], we subjected the analyses of our non-normal data also to non- parametric tests in order to further substantiate our findings. The interested reader may refer to S1 File in the supplementary material to review these results. In addition, a tau correlation was calculated between the difference-scores of subjective and physiological sexual arousal in order to assess whether sexual arousal changes (due to the manipulation) were similar in the subjective and physiological experience.

## Results

### Pre-existing group differences and manipulation check

Tests for possible pre-existing group differences and manipulations checks were performed before analysis. Table 1 illustrates the mean scores of the variables for which this was done, along with the variables of the main analysis, as a function of condition (experimental vs. control group), and Table 2 illustrates the scores of the variables from which the difference scores were calculated. Three participants that responded with a decrease in physiological arousal to the pornographic material were not considered in the analyses, as we would not be able to draw any conclusions from their data (n = 3; 2 from the control condition and 1 from the experimental condition).

**Table 1:** Descriptive statistics.

	Overall		Control group				Experimental group				Difference
	Mean	*SD*	*Mean*	*SD*	*Range*	*n*	*Mean*	*SD*	*Range*	*n*	*Sign*.
***Pre-existing differences***											
*Disgust propensity*^a^	16.61	3.09	15.94	2.72	10–21	34	17.18	3.29	10–24	40	.09
*Disgust sensitivity*^a^	11.35	3.31	11.06	2.98	6–18	34	11.63	3.61	6–22	40	.47
*Induction of physiological*^b^ *sexual arousal*	6.46	8.14	7.74	10.74	.02–44.55	30	5.21	4.20	0.06–13.15	31	.23
*Peak of subjective sexual arousal*^a^	48.28	26.06	51.69	23.65	1–92	32	45.55	27.83	0–99	40	.32
***Disgust manipulation***											
Difference scores of physiological disgust^c^	3.08	7.81	1.20	2.15	-.79–10.05	34	4.68	10.22	-1.82–51.08	40	.06
Difference scores of subjective disgust^a^	21.60	31.24	11.25.	22.14	-10.00–93.00	32	29.88	35.05	-27.00–100.00	40	.01*
***Analysis of sexual arousal***											
Difference scores of physiological sexual arousal (after–before odor)^b^	.63	4.15	1.81	3.84	-6.97–12.90	30	-.31	4.19	-15.63–8.86	38	.04*
Difference scores of subjective sexual arousal (after–before odor)^a^	-13.93	25.55	-7.25	21.37	-69–32	32	-19.28	27.56	-98.00–36	40	.05*

Difference scores reflect the increase or decrease of the specified variable over the mentioned time-period. Negative difference scores indicate a decrease of the value of the specified variable over time and positive difference scores indicate an increase over time. Due to list-wise deletions, variables that are difference-scores do not always correspond one to one with the raw scores mentioned in Table 2.

*Significant at the .05 level

** significant at .01 level. The superscripts a, b and c refer to

a) subjective experience measured on a 0–100 VAS

b) penile circumference in mm, and

c) μV.

**Table 2:** Descriptive statistics raw data.

	Control group				Experimental group			
	*Mean*	*SD*	*Range*	*n*	*Mean*	*SD*	*Range*	*n*
**Physiological sexual arousal in mm**								
Before the pornographic-video	64.88	7.74	55.10–90.11	26	55.95	6.09	49.86–80.06	32
During the pornographic-video	71.59	12.26	58.41–114.50	32	61.16	7.40	50.01–81.38	36
During the odor-induction	73.44	13.98	59.23–125.17	32	61.00	8.61	49.56–90.24	36
**Subjective sexual arousal**								
Before the manipulation^a^	41.12	24.79	1–79	33	42.61	25.87	0–92	40
After the manipulation^a^	34.88	21.31	0–79	33	24.68	23.43	0–76	40
**Physiological disgust (EMG)**								
Baseline disgust^b^	5.11	2.87	1.69–14.09	34	5.79	5.31	2.15–31.15	40
Disgust after first puff of odor^b^	6.31	4.06	2.09–17.33	34	10.47	11.50	2.69–56.90	40
**Subjective disgust**								
Before the manipulation^c^	6.18	15.03	0–62	32	7.20	11.34	0–48	41
After the manipulation^c^	18.06	24.28	0–94	32	37.08	33.60	0–100	41

^a^Subjective sexual arousal was measured directly after the manipulation, this includes an estimation of the level of sexual arousal before and an estimation of the level of sexual arousal after the odor-manipulation, on a 0–100 VAS

^b^ values in μV

^c^ Subjective disgust was measured on a 0–100 VAS.

We found no significant pre-existing group differences on disgust-propensity (*F* (1, 72) = 3.03, *p* = .09) or disgust sensitivity (*F* (1, 72) = .53, *p* = .47). The induction of sexual arousal was verified by pertinent t-tests. An overall increase in levels of physiological sexual arousal (from the neutral movie (M = 60.02mm) to the pornographic video (M = 65.92mm) was significantly different from zero (t (60) = 6.20, p < .01) with a mean increase of 6.46 mm in circumference (95% CI = 4.38–8.54). This confirmed that the pornographic video was, as intended, effective at inducing (genital) sexual arousal. Moreover, the groups were equally stimulated by the pornographic content before the odor was presented as evidenced by the lack of differences in the increase of physiological sexual arousal between the two groups (*F*(2, 59) = 1,49, *p* = .23; *M*_*contr*._ = 7.74; *M*_*exp*._ = 5.21). Similar to the genital sexual arousal, the subjective scores representing the peak level of subjective sexual arousal also deviated significantly from zero (t (60) = 6.20, *p* < .01; *M* = 48.28; *SD*_*=*_ 26.06; *95% CI* = 42.15–54.40) demonstrating a heightened level of subjectively experienced sexual arousal across the sample. Likewise, peak scores of subjective sexual arousal did not differ significantly between conditions (*F* (2,59) = 1.49, *p* = .23) thus indicating that the induction of sexual arousal was similarly effective for the experimental and the control group.

The induction of disgust by the aversive odor was verified by the subjective measurement of disgust. Difference scores of subjective disgust measures differed significantly between conditions (*F* (2, 70) 6.84; *p <* .05). With higher mean difference scores of disgust in the experimental condition (*M* = 29.88), compared to the control condition (*M* = 11.25), thus providing evidence for disgust induction. The physiological measurement of disgust also indicated a likely difference between the disgust experienced in the control and the experimental group. However, at *p* = 0.055 this difference was not significant. A greater disgust response was found in the experimental group (*M* = *4*.*68*), compared to the control group (*M* = 1.20). Lastly, it is interesting to note that in addition to these quantitative findings, appraisals made about the odor indicate a similar conclusion (Table 3).

**Table 3:** Interpretation of the odor.

	Experimental condition	%	Control condition	%
1	Rotten food / trash	23.08	Nothing	20.59
2	"Disgust"	12.8	Body odor / woman	20.59
3	Rotten eggs	12.8	Hospital / Ethanol	11.76

### Effect of aversive odor on sexual arousal

**Fig 1:**
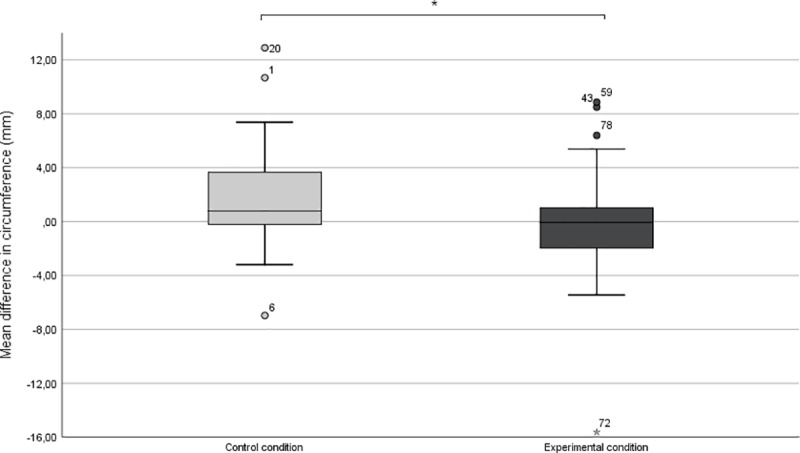
Boxplots depicting the change in circumference for physiological sexual arousal due to the aversive odor manipulation. These boxplots show the difference in circumference—from the period before to after the smell induction. The control condition (illustrated on the left) shows an increased penile circumference whereas in the experimental condition penile circumference is seen to stagnate or decrease.

**Fig 2:**
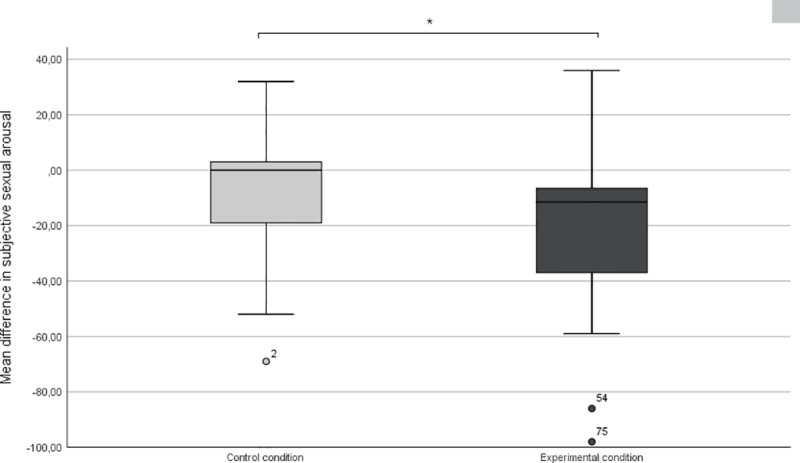
Boxplots depicting the change in subjective sexual arousal due to the aversive odor manipulation. The boxplots show the difference in the raw scores of subjective sexual arousal from the period before the smell induction to after the smell induction. The experimental condition on the right shows a greater decrease in subjective sexual arousal than the control condition on the left.

Two univariate ANOVA’s were performed to test the hypothesis that the aversive odor in the experimental condition was able to decrease (physiological and subjective) sexual arousal, relative to the odorless diluent in the control-condition. Providing evidence that the aversive odor indeed had an impact on lowering the physiological sexual arousal, a significant difference was found between the experimental condition (*M* = -.31 mm; *SD* = 4.19 mm) and the control condition (*M* = 1.81 mm; *SD* = 3.84 mm; *F* (2, 66) = 4.61; *p <* 0.05; *d =* 0.53; Fig 1). Difference scores of subjective sexual arousal (subjective baseline were assessed in retrospect) also differed significantly between conditions, (*F* (2.70) = 4.11; *p <* .05; *d =* 0.49; Fig 2) showing a greater average decline in sexual arousal in the experimental condition (*M* = -19.28; SD = 27.56) than in the control condition (*M* = -7.25; SD = 21.37). In addition, a small to moderately sized, correlation between the subjective and physiological difference score measures (rτ = .254**; p < .01) was found.

## Discussion

Based on the theory that disgust has the potential to interfere with the development and persistence of sexual arousal, in this study we tested the impact of an aversive odor on sexual arousal when men were already sexually aroused. The main result indicates that indeed subjective and physiological sexual arousal decreased significantly more in the aversive odor condition compared to the condition in which an odorless diluent was presented.

### The impact of odor on genital and subjective sexual arousal

Sexual arousal as measured by changes in penile circumference, declined or remained stable in the experimental condition, while it continued to increase somewhat in the control condition. The odor was strongly diluted and can only be considered a very mild disgust induction, as evidenced by the low score on the self-reported disgust (37 on a 100 point scale, See Results). So even despite this mild induction, it seems that disgust was able to, at least, attenuate a further increase in physiological sexual arousal when both the odor and a sexually arousing stimulus are presented simultaneously. Similarly, the odor also showed a negative effect on the levels of self-reported sexual arousal; which is expected given the congruency between subjective and physiological sexual arousal found in men [40].

These findings strengthen the view that a bidirectional relationship between disgust and sexual arousal applies also to men [11,13]. Furthermore, this study extends previous findings by showing the effect of disgust (even) on the penile response, which is indicative of reflexive responding [41]. Together, the subjective and physiological responses of sexual arousal show, that even when men are already in a sexually aroused state, disgust has an inhibitory influence on their sexual excitation. This finding might also have practical relevance; it suggests that disgust elicitors (e.g., odors) might be used to lower sexual arousal in the context of coercion. Inhibiting sexual arousal might be a way to prevent sexual coercion since sexual arousal has been linked to behavioral impulsive tendencies [16]. Following up on this, a start-up company (Invi bracelets) uses an aversive/disgusting odor encapsulated in a bracelet, as a means to safeguard potential victims of sexual coercion. It should be noted, however, that the current findings do not imply that the impact of disgust is restricted to its influence on sexual arousal but might affect other responses as well (e.g., individuals’ appetite for food items, [42]).

### Limitations

A number of limitations should be acknowledged. First, medical/ethical considerations required a high level of dilution of the odor. As a result, the experimental disgust induction was relatively weak. This may also explain why the mean difference in change of penile circumference between the two conditions was rather small. While such a small difference has been noted adequate in prior research (above 1 mm; [43]), the comparatively large variance in scores makes the outcome less firm. This also has relevance for the practical application of the odor. To know whether the effect of the odor on sexual arousal applies uniformly and to such a degree so as to bring about behavioral change, more research using stronger disgust elicitors is needed. Because presenting higher concentrations via an olfactometer might be hazardous, combining the odor with other sensory modalities such as disgusting sounds might be considered.

Second, some caution should be taken in generalizing these findings to the general male population. Specifically, the inclusion of penile measurements and pornography might have resulted in a selection bias, for example by causing more (sexually) inhibited individuals to refrain from volunteering in this study. Hence, in this hypothetical scenario the current sample might reflect relatively sexually assertive men; which could strengthen the practical relevance of the findings that even for these more assertive men, disgust can reduce sexual arousal. In the meantime, it can not be ruled out that potential sexual predators might have refrained from participation because they might have been afraid of being caught on the basis of their responding during the study. All in all, these types of sample biases need to be considered when interpreting these findings.

Third, participants were all young heterosexual students. Thus, our findings may not apply to people with a different age range, educational background or sexual orientation. On the other hand, as sexual functioning decreases with age [44], it seems plausible that an inhibition of sexual arousal would also occur in an older sample, yet further research in older participants is necessary to draw any firm conclusion in this respect.

Fourth, it should be noted that there were some missing data due to technical issues. However, since none of these problems can be attributed to or are an inherent effect of the variables, these missing data were treated as missing completely at random. Raw data is available on ‘*link to dataverse*’ for the interested reader.

Fifth, the participants were not asked about their level of experience with pornography and although participants were randomly assigned to conditions, it cannot be ruled out that groups had pre-existing differences in their exposure to pornography. However, sexual arousal was induced similarly in both groups.

Finally, it needs mention that although the efficacy of the manipulation seems to indicate that the reduction of arousal was mediated by increased (subjective) disgust, this relationship was not tested formally in the current study. It would be interesting in follow up research to have multiple measures of subjective disgust throughout the presentation of the arousing video—allowing to test whether indeed the reduction of arousal is preceded by heightened disgust.

### Future research leads

An important extension of the current findings would be to also study the impact of disgust on (impulsive) sexual approach behavior. Preliminary research already showed that sexually aroused individuals show a heightened approach tendency towards pictures of sexual penetration within the context of a computerized latency-based Approach Avoidance Task (AAT: [45])[46]. It would be important to test whether disgust can also attenuate these more impulsive approach tendencies in men; and whether such impact of disgust would be evident when men are already in a state of high arousal.

In addition, it would be important to examine whether disgust may also attenuate sexual approach behavior in an actual sexual context. A first step could be to use a scenario approach in which participants are asked about their inclination to show particular types of sexual approach behaviors in various types of situations under conditions of high vs. low disgust [13].

Moreover, the interpretation of the current results is restricted to the impact of an odor versus an odorless condition. To disentangle whether the reduction on physiological sexual arousal is driven by the selected odor or if it reflects a more general effect of odor-induced distraction, it is important for future research to more specifically test the influence of the disgusting odor in comparison to another perceivable odor of similar intensity but of a different valence. However, the control condition was (except for the nature of the smell) just as distracting as the odor condition, thus the difference we observe can be safely assumed to be due to the nature of the odor rather than simply being a distracting factor.

Finally, it needs mentioning that this study, as a first step to test whether the selected aversive smell would lower subjective and physiological sexual arousal, relied on mean values of the PCR during the critical stages of the experiment. It would be relevant as a second step to follow up this work with designs that allow to reliably test the pattern of responding over (exposure) time and to evaluate individual differences in the onset of responding, as well as dose-response relationships.

## Conclusion

In conclusion, the current findings strengthen the existing theory about the bidirectional relationship between disgust and sexual arousal in men [11,47]. Both subjective and physiological sexual arousal, were negatively affected by a disgusting/aversive odor of relatively mild intensity. The finding that a negative effect of disgust was also expressed/shown on the penile response, further strengthens the idea that disgust might hamper behavioral actions motivated by sexual arousal (e.g., poor judgment, coercive sexual behavior, etc.). Thus, these data suggest that an aversive odor is potent enough to restrict already present subjective and genital sexual arousal. This may also have societally relevant applications. For example, the current findings suggest that disgust-elicitors may be useful as a tool for self-defence against attempts of coercive sexual rapprochement.

## Supporting information

S1 Fig Procedural diagram. (DOCX)

S1 File Non-parametric test results. (DOCX)
