# Correction: The influence of olfactory disgust on (Genital) sexual arousal in men

**DOI:** 10.1371/journal.pone.0214330

**Published:** 2019-03-19

**Authors:** Charmaine Borg, Tamara A. Oosterwijk, Dominika Lisy, Sanne Boesveldt, Peter J. de Jong

In the Author Contributions, Peter J. de Jong (PjD) should be listed as one of the persons who contributed to conceptualization, formal analysis, investigation, methodology, project administration, resources, supervision, and writing–review & editing.
