# Supplementary figures and images for: The influence of olfactory disgust on (Genital) sexual arousal in men
Source: PLoS One. 2019 Feb 28;14(2):e0213059. doi: 10.1371/journal.pone.0213059 (PMC6394938; doi:10.1371/journal.pone.0213059)

#
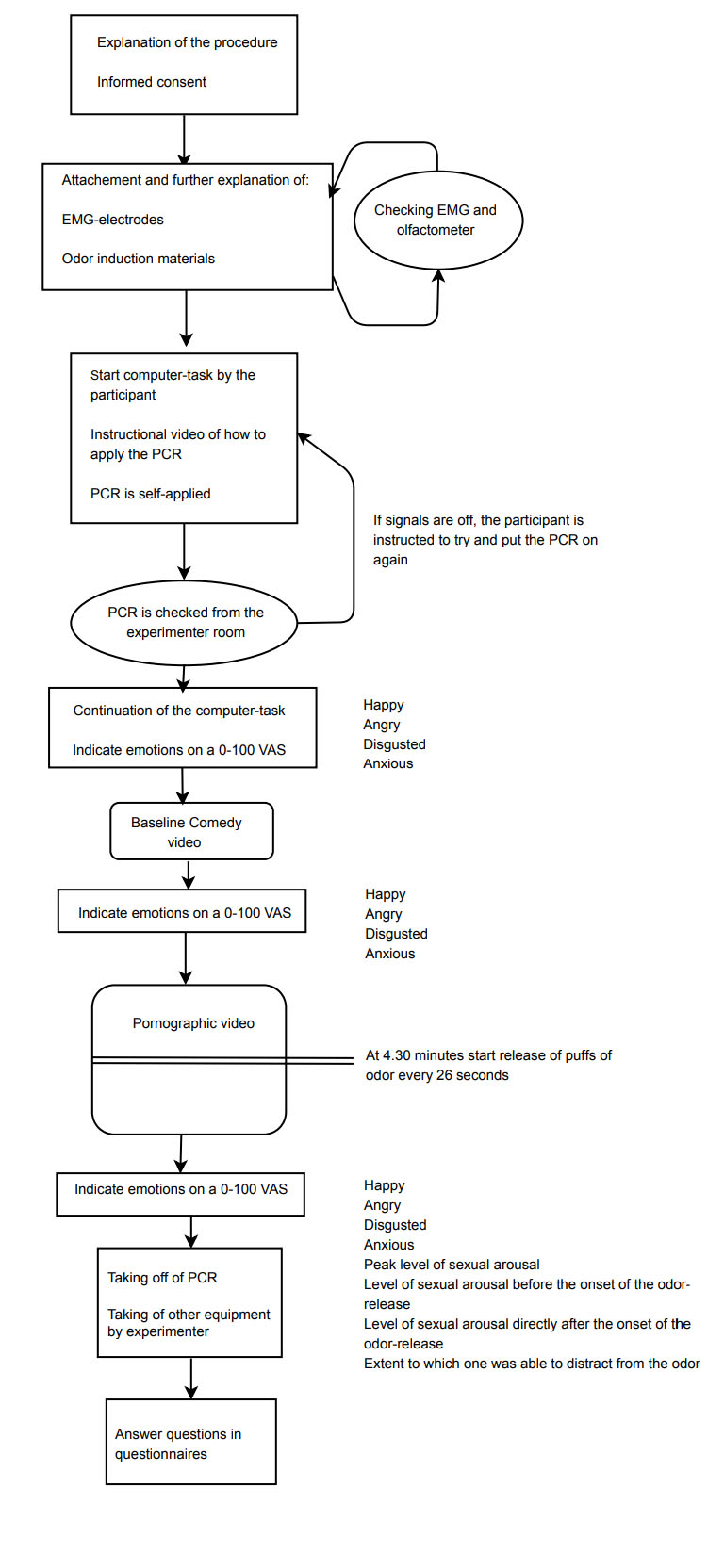
S1. Fig 1 Procedural diagram

Supplement: S1 Fig — (DOCX) [file pone.0213059.s001.docx]
