# Supplementary material for: The influence of olfactory disgust on (Genital) sexual arousal in men
Source: PLoS One. 2019 Feb 28;14(2):e0213059. doi: 10.1371/journal.pone.0213059 (PMC6394938; doi:10.1371/journal.pone.0213059)
Supplement: S1 File — (DOCX) [file pone.0213059.s002.docx]

**S2. Non-parametric test results**

As ANOVA’s and t-tests are considered robust against violations of normality to a high degree [1] and have a higher power than non-parametric tests (such as the Mann-Whitney U test [2] ANOVA’s were the preferred method of conducting our main analyses in the manuscript. In order to strengthen the findings for the variables that *did* possess violations of normality, we verified the results we found using ANOVA’s and t-tests by performing the equivalent non-parametric tests.

## *Manipulation check sexual arousal*

An overall increase in levels of physiological sexual arousal (from the neutral movie (*Mdn* = 64.50) to the pornographic video (*Mdn* = 68.39)) was verified by a Wilcoxon Signed-Ranks Test Z = -6.79, *p* <.01. This confirms that the pornographic movie was effective in inducing genital sexual arousal and corresponds to the t-test in the manuscript (t (60) = 6.20, *p* <.01). The subjective scores representing the peak level of subjective sexual arousal were also significantly different from zero (One-Sample Wilcoxon Signed-Ranks Test; Z = 7.32, *p* <.01; *Mdn* = 53.00), demonstrating a heightened level of subjectively experienced sexual arousal across the sample.

## *Differences between groups*

Differences between groups and their respective significance levels where calculated using a Mann Whitney U test are presented in S2 Table 1. This S2 Table 1 also provides the corresponding median values and a comparison of the significance levels of the Mann Whitney U to significance levels when using ANOVA’s.

Both tests can be seen to agree on which variables show a significant difference between groups. Thus strengthening our conclusions based on the ANOVA’s.

| **S2 Table 1, Comparison of parametric and non-parametric tests** | | | | | | | | |
| --- | --- | --- | --- | --- | --- | --- | --- | --- |
|  | Overall | | Control group | | Experimental group | | Difference | |
|  |  | |  | |  | | ANOVA | Mann Whitney U |
|  | Mean | *Median* | *Mean* | *Median* | *Mean* | *Median* | *Sign.* | *Sign.* |
| ***Pre-existing differences*** | | | | | | | | |
| *Induction of physiological^b^ sexual arousal* | 6.46 | 4.62 | 7.74 | 4.42 | 5.21 | 4.88 | .23 | .72 |
| *Peak of subjective sexual arousal^a^* | 48.28 | 53.00 | 51.69 | 56.00 | 45.55 | 49.00 | .44 | .32 |
| ***Disgust manipulation*** |  |  |  |  |  |  |  | |
| Difference scores of physiological disgust^c^ | 3.08 | .63 | 1.20 | .44 | 4.68 | 1.16 | .06 | .05 |
| Difference scores of subjective disgust^a^ | 21.60 | 7.5 | 11.25. | 1.50 | 29.88 | 20.50 | .01* | .01** |
| ***Analysis of sexual arousal*** | | | | | | | | |
| Difference scores of physiological sexual arousal (after – before odor)^b^ | .63 | .19 | 1.81 | .79 | -.31 | -0.07 | .04* | .02* |
| Difference scores of subjective sexual arousal (after – before odor)^a^ | -13.93 | -9.00 | -7.25 | .00 | -19.28 | -11.50 | .05* | .01** |
| Difference scores reflect the increase or decrease of the specified variable over the mentioned time-period. Negative difference scores indicate a decrease of the value of the specified variable over time and positive difference scores indicate an increase over time. Due to list-wise deletions variables that are difference-scores do not always correspond one to one with the raw scores mentioned in Table 2**.** *Significant at the .05 level, ** significant at .01 level, ^a^subjective experience measured on a a 0-100 VAS, ^b^ penile circumference in mm, ^c^ μV . | | | | | | | | |

## References

1. Blanca MJ, Alarcόn R, Arnau J, Bono R, Bendayan R. Non-normal data: Is ANOVA still a valid option? Psicothema. 2017; 29(4): 552-557. doi: 10.7334/psicothema2016.383.
2. Nachar N. The Mann Whitney U: A testfor assessing whether two independent samples come from the same distribution. 2008; 4(1): 13-20. doi: 10.20982/tqmp.04.1.p013.
